# 407. Cardiac Troponin I Levels Following mRNA-1273 Vaccination in Participants 12 Through 30 Years of Age: Results from a Phase 4 Randomized, Placebo-Controlled Study

**DOI:** 10.1093/ofid/ofaf695.014

**Published:** 2026-01-11

**Authors:** Susan Pfeiffer, Veronica Urdaneta, Xiaowei Wang, Bethany Girard, Frances Priddy, Margaret Rhee, Scott Striplin, Spyros Chalkias

**Affiliations:** Moderna, Inc., Cambridge, Massachusetts; Moderna, Inc., Cambridge, Massachusetts; Moderna, Inc., Cambridge, Massachusetts; Moderna, Inc., Cambridge, Massachusetts; Moderna, Inc., Cambridge, Massachusetts; Velocity Clinical Research, Cleveland, Ohio; Velocity Clinical Research, Cleveland, Ohio; Moderna, Inc., Cambridge, Massachusetts

## Abstract

**Background:**

Myocarditis is a very rare event following COVID-19 vaccination with the highest risk observed in males 12 through 24 years of age. It has typically been observed in the first week following vaccination, with resolution within a few days with conservative management. Cardiac troponin I (cTnI) is a serum biomarker for myocardial injury that is not specific to myocarditis and has not been studied extensively in younger people. Additionally, elevated cTnI levels may be associated with other cardiac and non-cardiac conditions, and cTnI levels after receipt of mRNA COVID vaccines are not well characterized. This study assessed cTnI levels following administration of mRNA-1273 KP.2 variant-containing vaccine, compared with placebo in participants 12 through 30 years of age.

**Methods:**

In this Phase 4, observer-blind, placebo-controlled, randomized study, 1,000 healthy participants at 24 U.S. sites were randomized to receive 50 µg of mRNA-1273.712 or placebo. Participants crossed over to receive an injection of the alternate product 28 days later. cTnI was assessed at baseline, 3 and 28 days after each injection. Elevated cTnI values were defined as > 53.53 pg/mL for males and 38.64 pg/mL for females according to the cTnI assay package insert. To aid in interpretation of cTnI results, participants recorded vigorous physical activity in an electronic diary. Safety was assessed throughout the study, and participants were followed for 28 days after the 2^nd^ injection.

**Results:**

mRNA-1273.712 was well tolerated with no new safety concerns identified. 981 participants had evaluable cTnI results. Most participants ( > 98%) had normal cTnI levels at all time points throughout the study. 18 out of 981 participants had at least 1 elevated cTnI result at any timepoint during the study. Of the 18, 7 had elevations at baseline prior to any study injection. Among the remaining 11 participants, 3 had elevated cTnI 28 days after receipt of mRNA-1273.712 and 8 had elevations following placebo. 11 of the 18 participants with elevated cTnI reported recent physical activity and none exhibited clinical signs or symptoms of myocarditis or pericarditis.

**Conclusion:**

Vaccination with mRNA-1273 in adolescents and young adults was not associated with elevated cTnI.

**Disclosures:**

All Authors: No reported disclosures

